# Influence of prior knowledge on eye movements to scenes as revealed by hidden Markov models

**DOI:** 10.1167/jov.23.10.10

**Published:** 2023-09-18

**Authors:** Marek A. Pedziwiatr, Sophie Heer, Antoine Coutrot, Peter J. Bex, Isabelle Mareschal

**Affiliations:** 1 School of Biological and Behavioural Sciences, Queen Mary University of London, London, UK; 2 Univ Lyon, CNRS, INSA Lyon, UCBL, LIRIS, UMR5205, F-69621 Lyon, France; 3 Department of Psychology, Northeastern University, Boston, MA, USA

**Keywords:** eye movements, prior knowledge, scene perception, individual differences

## Abstract

Human visual experience usually provides ample opportunity to accumulate knowledge about events unfolding in the environment. In typical scene perception experiments, however, participants view images that are unrelated to each other and, therefore, they cannot accumulate knowledge relevant to the upcoming visual input. Consequently, the influence of such knowledge on how this input is processed remains underexplored. Here, we investigated this influence in the context of gaze control. We used sequences of static film frames arranged in a way that allowed us to compare eye movements to identical frames between two groups: a group that accumulated prior knowledge relevant to the situations depicted in these frames and a group that did not. We used a machine learning approach based on hidden Markov models fitted to individual scanpaths to demonstrate that the gaze patterns from the two groups differed systematically and, thereby, showed that recently accumulated prior knowledge contributes to gaze control. Next, we leveraged the interpretability of hidden Markov models to characterize these differences. Additionally, we report two unexpected and interesting caveats of our approach. Overall, our results highlight the importance of recently acquired prior knowledge for oculomotor control and the potential of hidden Markov models as a tool for investigating it.

## Introduction

Individuals use knowledge about recently passed events to guide their behavior. For example, we do not touch a dish knowing that it has just been taken out of a hot oven. Although situations in which knowledge accumulation occurs are omnipresent, it remains unclear how this recently accumulated knowledge (what we call prior knowledge) guides further information-seeking. In particular, relatively little is known about how it affects how humans acquire visual information by means of shifting their gaze. To examine this issue, we conducted an eye-tracking study in which we used unique stimuli: sequences of static film frames that allowed for the accumulation of knowledge about unfolding events.

Typical experimental procedures used to study visual exploration involve presenting participants with sequences of images that are unrelated to each other. These procedures are usually conducted in laboratory conditions and, as a consequence, guarantee good control over most aspects of the experiments. They are also versatile, because they allow for presenting almost any set of images as stimuli. Owing to these features, these procedures have been used to tackle a wide range of research problems. To name a few, they allowed to characterize factors influencing oculomotor behavior while viewing emotionally laden images (Pilarczyk, Schwertner, Wołoszyn, & Kuniecki, 2019; Pilarczyk, Kuniecki, Wołoszyn, & Sterna, 2020; Pilarczyk & Kuniecki, 2014) or images depicting people (Birmingham, Bischof, & Kingstone, 2008; Cerf, Paxon Frady, & Koch, 2009; End & Gamer, 2017; Flechsenhar & Gamer, 2017), as well as to uncover idiosyncrasies in gaze behavior of individuals (Broda & de Haas, 2022; De Haas, Iakovidis, Schwarzkopf, & Gegenfurtner, 2019; Linka & de Haas, 2020; Zangrossi, Cona, Celli, Zorzi, & Corbetta, 2021) and create robust methods of predicting fixation locations (Kümmerer, Bethge, & Wallis, 2017; Kümmerer, Wallis, Gatys, & Bethge, 2022).

An assumption embedded in the procedures described is that viewing any image is independent of viewing the other images. Participants are usually shown images presented in random order to ensure that this assumption is met. Consequently, typical image viewing experiments do not allow for investigating the influence of recently acquired knowledge on oculomotor behavior. In these experiments, participants do not have the opportunity to accumulate information relevant to upcoming images and, therefore, view each image without prior knowledge about its content. This situation starkly contrasts with a typical real world experience, where the visual input is usually continuous—people observe events as they unfold, so the current visual input is usually related to the input that immediately preceded it. This continuity enables the accumulation of information about the current environment and what is happening with it. As a result, almost all visual input the individuals receive is embedded in prior knowledge relevant to it. However, owing to the above-mentioned methodological reasons, the consequences of possessing that knowledge for oculomotor behavior during static image viewing remain underexplored. Only a handful of studies have investigated them, and they either used contrived stimuli (Król & Król, 2018; Pedziwiatr, von dem Hagen, & Teufel, 2023) or relied only upon a very limited number of images (Dorr, Martinetz, Gegenfurtner, & Barth, 2010; Hutson, Chandran, Magliano, Smith, & Loschky, 2022). What these studies have in common is that they investigated how the gaze is affected by information that is inaccessible via eye movements at a given moment. This property distinguishes them from the typical image viewing experiments in which information accumulation occurs exclusively while viewing a particular image. For example, in a visual search task, participants may inspect a certain location and determine that it does not contain the target, accumulate that information, and inspect this location again if they wish to. This possibility to acquire previously accumulated information again by re-inspecting an image region makes that kind of knowledge accumulation distinct from the kind we are interested in here.

Nevertheless, there are at least two important strands of research that touch on how the different types of continuity of visual input—a necessary condition for knowledge accumulation—affect eye movements. The first strand investigates real-world tasks that extend over time, such as putting up a tent (Sullivan et al., 2021), making a sandwich (Hayhoe, Shrivastava, Mruczek, & Pelz, 2003), or walking in various settings (Bonnen et al., 2021; Ghiani, Van Hout, Driessen, & Brenner, 2023; Matthis, Yates, & Hayhoe, 2018; Patla & Vickers, 2003; Rothkopf, Ballard, & Hayhoe, 2016). These studies highlighted the interactions between eye movements and processes that require knowledge accumulation, such as planning, representing the environment, manual actions, or navigation (Tatler & Land, 2011). The second strand of research that explicitly acknowledges the importance of continuity of visual input explores how people process and understand visual narratives, such as comics (Foulsham & Cohn, 2021; Foulsham, Wybrow, & Cohn, 2016; Hutson, Magliano, & Loschky, 2018; Kirtley et al., 2018) and films (Kirkorian & Anderson, 2018; Loschky, Larson, Magliano, & Smith, 2015, Loschky, Larson, Smith, & Magliano, 2020; Rider, Coutrot, Pellicano, Dakin, & Mareschal, 2018; Smith, 2012; Valuch, König, & Ansorge, 2017; Wang, Freeman, Merriam, Hasson, & Heeger, 2012). In these media, a high-level narrative continuity is ensured by the plot and, especially in film, preserved even in the presence of abrupt changes in visual input, such as cuts and camera position shifts. The studies cited above demonstrated that following a plot—which requires accumulating information—has consequences for the oculomotor behavior of viewers.

Both these research strands demonstrate that prior knowledge matters for how visual input is sampled using eye movements. However, they both rely on very different stimuli and/or experimental settings than typical image viewing experiments. Therefore, relating findings from these two strands to the experiments that rely on viewing static images is nontrivial. To illustrate, consider that real-life tasks usually require manual action (which results in visible changes in the environment), often accompanied by head or body motion (which changes the available field of view), so in this case participants do not inspect the environment as a fixed static snapshot that would be equivalent to a single static image displayed in a laboratory-based experiment. Comics, in turn, usually consist of simplified drawings that contain specific graphical elements (e.g., speech balloons) across multiple panels and, therefore, are considerably different from typical scenes. Finally, films are obviously different because they contain motion. Overall, it is challenging to directly relate insights into the role of prior knowledge gained from the experiments on real-world tasks, comics, and films to static image viewing. Hence, the effects of recently gained prior knowledge manifesting in this context remain underexplored.

To examine these differences, we recently created a unique stimulus set comprising 80 sequences of static frames from films directed by Alfred Hitchcock (Pedziwiatr, Heer, Coutrot, Bex, & Mareschal, 2023). The sequences were arranged in such a way that participants viewed identical frames after acquiring knowledge that was either relevant to the content of these frames or not. Thus, we retained the well-controlled nature of typical image viewing experiments while creating conditions that enabled knowledge accumulation. We found that participants who had accumulated relevant prior knowledge exhibited less exploratory gaze behavior than those who did not. Our conclusion was based on the analysis of seven characteristics of eye movements: the number of fixations, average fixation duration, average interfixation distance, interobserver consistency, the probability of blinking, first saccade latency, and heatmap entropy. This fine-grained approach, however, had its limitations. First, all of our metrics largely ignored the temporal order of fixations. Each scanpath (i.e., a raw gaze trace of an individual participant on an individual frame) generated in a 2-second trial was collapsed over time into one number per metric (e.g., average fixation duration). Second, our metrics were decoupled from the spatial distributions of fixations on the frames. For example, they all could have identical values for two sets of fixations inspecting different regions of a frame. Third, relying on the metrics requires extracting fixations from the raw eye trace. This step involves researcher degrees of freedom (Simmons, Nelson, & Simonsohn, 2011; Wicherts et al., 2016), that is, analytical decisions, such as selecting a fixation extraction algorithm out of many available (Birawo & Kasprowski, 2022) and fine tuning its parameters, which might inadvertently affect the results. Fourth, although our approach was well-suited for testing hypotheses, it left little space for the exploration of the spatiotemporal structure of the rich data we collected.

To bypass these limitations, we analyzed the same data using a very different approach. Specifically, we modeled individual scanpaths as hidden Markov models (HMMs) and applied machine-learning techniques to them. An HMM is a mathematical construct describing—in a probabilistic fashion—a temporal evolution of a system with a finite number of possible states. It generates probable trajectories of the system's behavior that are expressed as the sequences of system's states. Modelling a scanpath as an HMM involves assuming that each data sample belongs to one state and finding HMM's parameters for which the HMM is most likely to generate the sequence of states constituting the scanpath. Methods based on this modelling have proven insightful and are commonly used in various research contexts (Chuk, Chan, & Hsiao, 2014; Coutrot, Binetti, Harrison, Mareschal, & Johnston, 2016; Coutrot, Hsiao, & Chan, 2018; Haji-Abolhassani & Clark, 2014; Hsiao, Lan, Zheng, & Chan, 2021, Hsiao, Liao, & Tso, 2022; Liu et al., 2013).

Using the HMMs has several advantages over the more traditional approach based on multiple oculomotor metrics we have used previously. First, HMMs offer a succinct way to holistically capture both the spatial and temporal dynamics of a scanpath. Second, analyses combining HMMs and machine learning algorithms (classifiers) offer data-driven ways of measuring the relative importance of different aspects of scanpaths for distinguishing between conditions in which they were recorded (e.g., see Coutrot et al., 2018). Third, HMMs can be analyzed in multiple ways, which facilitates sophisticated data exploration. Fourth, HMMs can be fit to raw data, which eliminates the researcher aforementioned degrees of freedom related to fixation extraction. However, using HMMs does not eliminate the problem of these degrees of freedom completely. Therefore, we have guarded against this concern by preregistering the general outline of our HMMs analysis.

To foreshadow our results, we found that an off-the-shelf linear classifier could distinguish between HMMs fitted to scanpaths of participants who viewed identical frames in different conditions: either with or without prior knowledge relevant to the frames’ content. An exploratory analysis of the classification results revealed the link between this content and the variability in eye movements along the horizontal dimension to which the classifier was sensitive. In addition to these main results, we also describe two methodological caveats we have encountered when working with HMMs: one related to the role of data dimensionality reduction in the classification analysis and one related to the influence of a random number generator on the outcomes of the HMMs fitting procedure. To our knowledge, these caveats have not yet been described in the literature on modelling gaze patterns with HMMs.

## Methods

### Stimuli, experimental design, and eye movements data

All analyses reported here were conducted on data from a study described in our earlier article (Pedziwiatr, Heer et al., 2023), and full details of how these data were collected can be found there. The preregistration of our study (outlining the HMMs analysis reported here), the data, and a script for downloading our stimuli are available at the following link: https://osf.io/et7mr/?view_only=6f86dc8211d845c7b2c09ef6f45baf64.

**Figure 1.**
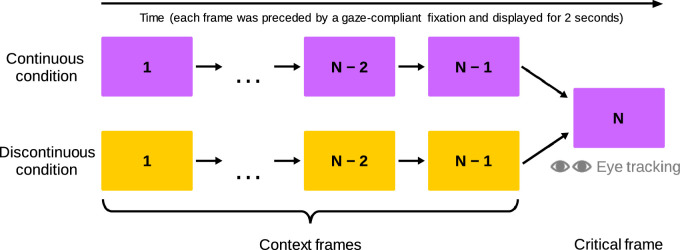
Schema of the experimental procedure. Participants viewed N-element sequences of film frames (*N* varied between trials; in the Figure, different colors indicate different films). Depending on the condition, frames from 1 to *N* – 1 either preceded the *N*th frame in the film from which it was extracted (continuous condition) or originated from a different film (discontinuous condition; see colors in the Figure). Consequently, participants in the continuous condition viewed the final frames of the sequences, called the critical frames, while having knowledge of events that led to the situations shown in them, whereas participants in the discontinuous condition had knowledge of different, unrelated events. In our analyses, we compared the eye movements registered on the critical frames in these two conditions to reveal the influences of prior knowledge on gaze behavior.

The stimuli set in our study consisted of 80 sequences of static frames extracted from films directed by Alfred Hitchcock. It was manually assembled from frames available on the 1000 Frames of Hitchcock project website (https://the.hitchcock.zone/wiki/1000_Frames_of_Hitchcock). The frames in the sequences were spaced several seconds apart. In each sequence, the final frame was called the critical frame, while all frames preceding it—the context frames. Our experimental procedure had two conditions which differed only in the context frames; the critical frames were identical in both (Figure 1). The two contexts paired with a given critical frame always had the same lengths, but these lengths differed for different critical frames (mean length was 5.35 frames, with a standard deviation of 1.16 frames). In the continuous condition, the context frames were extracted from the same film as the critical frame and depicted a course of events from which the situations depicted in the critical frames naturally followed. In the discontinuous condition, the context frames depicted a coherent course of events but were extracted from a different film and were unrelated to the critical frame's content. As a consequence, participants viewing the context frames in both conditions accumulated knowledge about the unfolding events. However, this knowledge was relevant to the situation depicted in the critical frames only in the continuous condition. Therefore, in our study, different groups of participants viewed identical critical frames while possessing prior knowledge that was either relevant or irrelevant to the frames’ content. We were interested in comparing gaze patterns on these frames between the two groups.

We conducted two similar experiments that relied on this concept. We focus on the results of experiment 1; results of key analyses for data from experiment 2 are reported in the Main results for data from experiment 2. Participants contributed data to our dataset from experiment 1. All were instructed to carefully look at the images to be presented and then viewed our sequences. Each participant viewed all 80 critical frames (one-half in each condition, counterbalanced between groups). We discarded 1.25% of that data based on prespecified criteria (see Pedziwiatr, Heer et al., 2023 for details). We presented an attention check question after each critical frame to keep the participants engaged. The questions pertained to the frames’ content and were tailored for each frame individually. All frames were presented for 2 seconds, had a size of 28.4° × 16.0° of visual angle, and were preceded by a gaze-compliant fixation dot. Throughout the procedure, participants’ eye movements were recorded using a Tobii 4c eye tracker with a sampling frequency of 90 Hz. Analyses we report here pertain only to gaze traces recorded on the critical frames.

### HMMs

**Figure 2.**
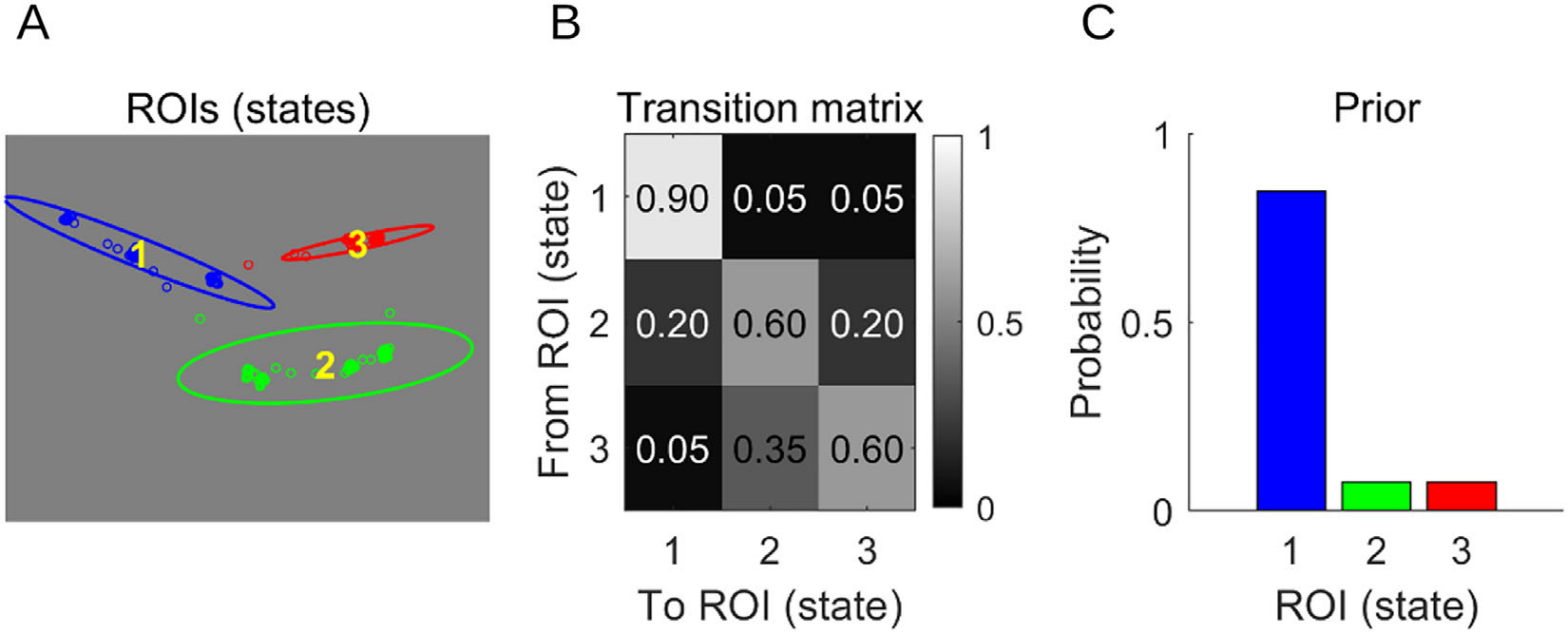
Elements of an HMM fitted to an individual scanpath. (**A**) ROIs corresponding to three states (ellipses) and a modelled scanpath consisting of individual samples registered by an eye tracker (dots). (**B**) Transition matrix, determining the probabilities of transitioning between each two states. High values at the diagonal indicate that at any given time, staying in the same state (fixating the ROI) is more probable than moving to a different state (making a saccade). (**C**) Prior, determining for each state the probability that a sequence of states generated by the HMM starts from it. Note that this Figure presents a contrived illustration, not an HMM fitted to real data. HMM = hidden Markov model; ROI = region of interest.

We modelled individual scanpaths from our data as HMMs. An HMM is a type of Markov model—a mathematical construct often used for modelling systems that switch between several different states over time. A Markov model consists of three elements (Figure 2). Here, they all have interpretations specific to our application of these models. First, states—a set of regions of interest (ROIs) within an image. Changes of an HMM's state correspond to gaze shifts between these ROIs. Second, a transitions matrix—a matrix specifying the probabilities of transitioning between states. It is assumed that at each transition, the next state depends only on the current state and not on the past states. This feature of these models is called the Markov property. Third, a prior—a probability distribution over states specifying how likely each of them is to be an initial state of the model. In other words, the prior specifies for each ROI (state) its chance of being attended first. The difference between the Markov model and a HMM is that in the latter, the states are not observable directly. Instead, they are inferred from the data and modelled as probability distributions. The process of fitting an HMM to a scanpath assumes that each point belonging to the scanpath has originated from one of these distributions (states) that are not accessible directly. This difference is important from a mathematical standpoint. However, for the sake of simplicity, we equate the states with ROIs in the descriptions of our analyses provided here.

To model our scanpaths as HMMs, that is, find HMMs that were most likely to generate the scanpaths recorded on the critical frames, we used the *SMAC with HMM* toolbox (Coutrot et al., 2018; see also Chuk et al., 2014). This toolbox first uses a variational approach (McGrory & Titterington, 2009) to determine ROIs constituting states. This approach assumes that each state is a two-dimensional Gaussian distribution and determines the values of the four parameters defining it: two coordinates of its center (horizontal and vertical) and two coefficients of its symmetrical covariance matrix. The remaining parameters of an HMM are determined once the states are known.

We set the number of states in each HMM to three, following Coutrot et al. (2018). This resulted in each of our HMMs being a vector of 24 numbers: 6 coordinates of state's centers, 6 coefficients of states’ covariance matrices, three values for a prior, and 9 coefficients of a covariance matrix. Instead of providing the number of states a priori, we could have also determined it in a data-driven way for each scanpath (see Coutrot et al., 2018 for details). However, preliminary data analyses indicated that, although this solution would make our analyses significantly more complicated, it was unlikely to provide additional insight into our data or lead to qualitatively different results. Therefore, we discarded it.

### Classification analysis

We expected that if the gaze patterns registered on the critical frames differed between the continuous and discontinuous conditions, then the HMMs fitted to the scanpaths would capture these differences and, consequently, differ between the conditions as well. To test this prediction, we used a supervised machine-learning approach. Broadly speaking, such approaches assume that if two sets of data samples belong to different categories (classes) that differ from each other in a systematic fashion, a classifier should be able to capture these differences (for an extensive overview, see Hastie, Tibshirani, & Friedman, 2009). A typical analysis based on this approach has two phases. First, the classifier is trained—it processes samples for which the class is known and attempts to learn to distinguish between different classes. Second, novel samples are presented to the classifier—if it has learned the properties differentiating between the classes, it should correctly assign the novel samples to their classes. Methods based on machine learning often offer an attractive alternative to more traditional analytical approaches and have already been proven useful for providing insights about human oculomotor behavior (e.g., see Borji & Itti, 2014; Coutrot et al., 2016; and Kümmerer et al., 2017).

Here, we tested whether an off-the-shelf classifier could correctly assign the HMMs to the two conditions at the above-chance level. Given that we were interested in the effects of prior knowledge that manifest as differences in scanpaths registered on the same frames, we did this for each critical frame separately. Our classification analysis closely followed an approach introduced by Coutrot et al. (2018) and largely relied on MATLAB code from their *SMAC with HMM* toolbox. We used a default classifier from this toolbox—a linear classifier based on the linear discriminant analysis. We chose it because it is simple and does not require adjusting any parameters. Before being passed to the classifier, all values constituting the HMMs for a given critical frame were first normalized to have zero mean and unit standard deviation (within each condition separately) and then jointly regularized with the parameter lambda set to 0.00001 (see Coutrot et al., 2018 for details).

To assess how well the HMMs could be classified, we relied on a leave-one-out cross-validation procedure. Specifically, we designated each HMM, in turn, as left out, trained the classifier on the remaining HMMs (each time anew), and then tested if that newly trained classifier could correctly classify the left-out HMM. The proportion of left-out HMMs classified correctly amounted to the classifier accuracy, which served as a measure of classifier performance for a given critical frame. This performance was always compared with a chance level performance, that is, the accuracy expected when the data used to train the classifier did not contain information that allowed to distinguish between the conditions. To calculate the chance level performance values for each critical frame, we randomly shuffled the pairing of HMMs and conditions from which their corresponding scanpaths originated and then conducted the cross-validation again. Repeating this procedure 2,000 times resulted in 2,000 chance level performance values for each critical frame. We averaged these values per frame to derive the chance level accuracies.

Note that, in each of the 2,000 repetitions of the above-mentioned procedure, we obtained a set of 80 chance level values (one per critical frame). Averaging these values per set resulted in 2,000 values of average (for all the frames) classifier performance in cases in which the pairing between HMMs and conditions was random within each frame. These 2,000 values served as a null distribution for a permutation test we conducted to assess the statistical significance of our classification results at the level of the whole dataset (similarly as in Coutrot et al., 2018). Specifically, we calculated a *p* value for the mean classification accuracy as a fraction of all values from the null distribution that were higher than this mean. In this test, we adopted 0.05 as the threshold of statistical significance.

## Results

### Experiment 1: Main results

**Figure 3.**
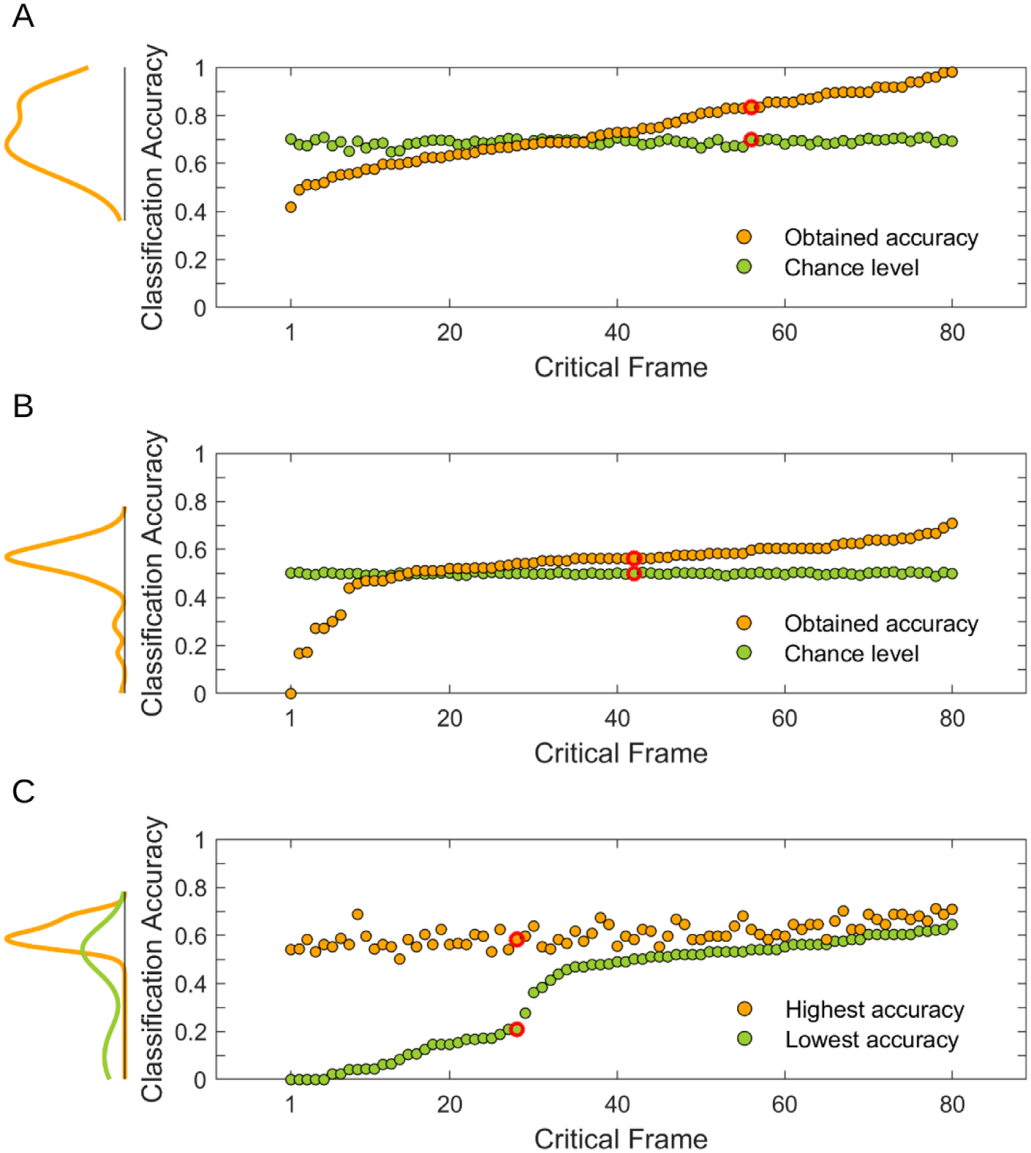
Results from the different variants of the classification analysis. Dots on all panels represent individual critical frames. The results for HMMs before and after their dimensionality has been reduced by retaining only their first principal component are shown on, respectively, (**A** and **B**). Notice that the dimensionality reduction leads to lower chance levels and an increase in the percent of frames classified above chance. For details, see the section HMMs and classification: Caveat one—chance levels. (**C**) For each frame, the highest and the lowest classification accuracy obtained in 10 repetitions of the classification analysis, where each repetition was conducted on HMMs fitted after initializing the random number generator with a different seed (number). For details, see the section HMMs and classification: Caveat two—a random number generator. Note that the ordering of frames is different on each panel: on (**A** and **B**), they are ordered by the increasing obtained accuracy, whereas on (**C**) they are ordered by the increasing lowest accuracy. Red circles on all panels indicate results for the same critical frame. On the left-hand side of each panel, marginal density plots are presented. HMM = hidden Markov model.

We found that our classifier could distinguish between HMMs fitted to scanpaths registered in different experimental conditions better than at chance (obtained accuracy: *M* = 73.93%, *SD* = 13.64; chance level accuracy: *M* = 68.85%, *SD* = 1.28; percent of frames classified above chance: 56.25%) (Figure 3A). This result was statistically significant (*p* < 0.001) and indicated that HMMs captured information differentiating scanpaths from the different conditions.

While working on this analysis, we noticed two phenomena that are worth highlighting because they constitute caveats one needs to be mindful of when using HMMs. First, our chance level classification accuracies were surprisingly high. Second, the seed of the random number generator set before fitting the HMMs often had large downstream effects on the classification results. We dedicate the subsequent two sections to these caveats. To the best of our knowledge, these have not been described previously in the literature on using HMMs to model scanpaths.

#### HMMs and classification: Caveat one—chance levels

To illustrate this caveat, consider the critical frame marked on all panels of Figure 3 with a red circle. The classification accuracy for this frame amounted to 83.33% (see Figure 3A). Given that for this frame, the number of HMMs was equal in both conditions, a theoretical chance level classification accuracy for it was 50%. Based on these two pieces of information, one could judge that the classifier performed reasonably well. However, the empirical chance level that we calculated (see the Classification analysis section) was 69.93%—almost 20% higher than the theoretical 50%, which changes the evaluation of the classifier performance. This phenomenon, although explained within the context of a single frame, was pervasive throughout our dataset (Figure 2A). We hypothesized that it stemmed from a high feature-to-sample ratio (Vabalas, Gowen, Poliakoff, & Casson, 2019, esp. Figure 6; see also Combrisson & Jerbi, 2015). Recall that each HMM constituting an individual sample in our classification procedure consisted of 24 numbers, and each number was a value of a single feature. Given that 48 scanpaths were registered on the frame considered here (and, in consequence, 48 HMMs were fitted), the feature-to-sample ratio for that frame amounted to ½. It is known that feature-to-sample ratios of that magnitude lead to discrepancies between theoretical chance levels and empirical chance levels (Combrisson & Jerbi, 2015; Vabalas et al., 2019).

To confirm empirically that the high chance levels resulted from the high feature-to-sample ratios, we reduced the dimensionality of our data and conducted the classification analysis again. The reduction lowered the feature-to-sample ratios (because it decreased the number of features while preserving the number of samples), and consequently, we expected to observe lower chance levels. To perform the reduction, we used principal component analysis (implemented in Matlab function *pca*). We applied it separately to each set of HMMs fitted to scanpaths registered on a single critical frame. Then, in each set, we discarded all principal components but the first. Thereby, we reduced the number of values describing an individual HMM from 24 (Figure 2) to one. The first principal components we retained could, on average, explain 40.21% of the variance (*SD* = 7.85) in the data.

In line with our expectations, this time, the classification analysis yielded lower chance levels, as well as lower accuracies (obtained accuracy: *M* = 53.84%, *SD* = 11.85; chance level accuracy: *M* = 49.88%, *SD* = 0.42) (Figure 3B). The average decrease in the chance level between the full data and the dimensionality-reduced data amounted to 18.97% (*SD* = 1.4), whereas the average decrease in the obtained accuracy—to 20.09% (*SD* = 17.85). The classification result remained statistically significant (*p* = 0.008) and interestingly, the percent of frames for which the obtained accuracy exceeded the chance level increased (from 56.25% for the data before the dimensionality reduction to 82.5% here).

#### HMMs and classification: Caveat two—a random number generator

The second caveat we want to highlight pertains to the dependence of the HMM-fitting procedure—and, in consequence, results of the classification analysis—on an algorithm generating random numbers used in this procedure. Computer-generated random numbers are, in fact, pseudo-random (L'Ecuyer, 2012). That is, they meet the mathematical criteria of being random but are generated by a deterministic algorithm. In most programming languages, such algorithms are called random number generators, irrespective of their specifics. Many, if not all, of these generators require initialization with a number called the seed. For a given seed, they always produce the same sequences of (pseudo)random numbers. Here, we observed that initializing a random number generator with different seeds and then fitting HMMs to the scanpaths resulted in different HMMs each time. This observation was perhaps unsurprising, given that the procedure of fitting the models relies on this generator. What was surprising, however, was the magnitude of the downstream effects the seed had on the classification results. We discovered this phenomenon by accident and investigated it systematically afterward.

To this end, we fitted HMMs to our scanpaths 10 times, each time initializing the random number generator with a different seed (using the Matlab function *rng* with default settings that indicated using Mersenne Twister generator). We made an arbitrary choice to use the first 10 unique Fibonacci numbers as the seeds. Having obtained 10 distinct sets of HMMs, we conducted the classification analysis with dimensionality reduction described in the previous section on each set separately.

The summary results of these 10 analyses are reported in Table 1. We observed that mean chance level classification accuracy and median obtained classification accuracy remained stable across the 10 repetitions, while mean obtained accuracy and the percent of frames classified above chance were more variable. This variability was accompanied by differences in the outcomes of the statistical test—the results were statistically significant in seven out of 10 cases (see the *p* value for the mean column).

**Table 1.** Results of the classification analysis with dimensionality reduction conducted on the 10 sets of HMMs fitted to the same scanpaths but differing regarding the seed of a random number generator used in the fitting procedure. IQR = interquartile range; *SD* = standard deviation. Each *p* < 0.05 is printed in bold.

Seed	Mean obtained classification accuracy (*SD*)	Mean chance level classification accuracy (*SD*)	Median obtained classification accuracy (IQR)	Percent of frames classified above chance	*p* value for the mean
0	50.29 (17.05)	49.93 (0.38)	56.25 (10.42)	75	0.421
1	52.8 (14.88)	49.99 (0.37)	56.25 (8.29)	78.75	**0.042**
2	51.94 (15.23)	49.97 (0.36)	55.9 (10.42)	77.5	0.124
3	53.49 (12.44)	49.93 (0.37)	56.25 (7.87)	81.25	**0.013**
5	54.3 (10.31)	49.92 (0.4)	56.25 (7.98)	78.75	**0.002**
8	53.18 (13.57)	49.92 (0.35)	55.9 (8.84)	81.25	**0.019**
13	54.08 (12.55)	49.95 (0.36)	56.25 (8.33)	83.75	**0.003**
21	52.26 (14.83)	49.97 (0.4)	56.25 (8)	81.25	0.084
34	53.66 (13.2)	49.92 (0.38)	56.39 (8.33)	83.75	**0.011**
55	53.2 (14.2)	49.96 (0.39)	56.25 (9.35)	83.75	**0.021**

Additionally, for each critical frame, we compared the highest and the lowest classification accuracy obtained in the 10 repetitions of the classifications analysis (see Figure 3C). This comparison revealed that the lowest accuracies (*M* = 37.72%; *SD* = 22.1) varied between frames much more than the highest accuracies (*M* = 60.72%; *SD* = 4.92).

#### Loadings of the first principal components: Analysis and interpretation

Given that the first principal component alone was sufficient for classifying our scanpaths to the two conditions with the above-chance accuracy, we investigated what information was carried by it. This information was likely crucial for the classification accuracy and, therefore, provided insights into the nature of the differences in gaze behavior between our conditions. Importantly, these insights are complementary to the insights gained in our previous article (Pedziwiatr, Heer et al., 2023) because here, owing to the interpretability of the HMM coefficients, they would take the spatiotemporal properties of scanpaths and their relationship to the frames’ content into account.

However, retrieving any information from the principal components first required deciding on the data from which to retrieve it, that is, selecting a specific set of HMMs for each critical frame out of the 10 we fitted using different seeds of a random number generator. We already knew that this selection might affect our findings, so we conducted it in a principled manner. Specifically, we guided it using our previous results and for each frame we opted for the HMMs fitted using the seed for which the classification accuracy was the highest. Therefore, here, the HMMs we selected for different frames could be fitted using any 1 of the 10 seeds, but all HMMs fitted to scanapths registered on a given frame were always fitted using the same seed. We decided to select these HMMs because we assumed that they best captured the differences between conditions within each frame.

Recall that each HMM consists of 24 numbers (coefficients) that define its three elements (Figure 2): ROIs corresponding to the states (each was defined by the coordinates of its center and the coefficients of its covariance matrix), a transition matrix, and a prior. Calculating the principal components of a set of HMMs involved calculating the weights given to each HMM coefficient in each component. These weights are called “loadings.” The absolute value of a given loading determines the importance of its corresponding coefficient to a given principal component. Therefore, the analysis of loadings can reveal which coefficients are responsible for the majority of variance in the data—the loadings of these coefficients have high absolute values in the initial principal components. Given that the loadings for each critical frame were calculated for HMMs fitted to scanpaths from both conditions, the variability in the data they characterize is expected to stem from the differences in eye movements between conditions.

**Figure 4.**
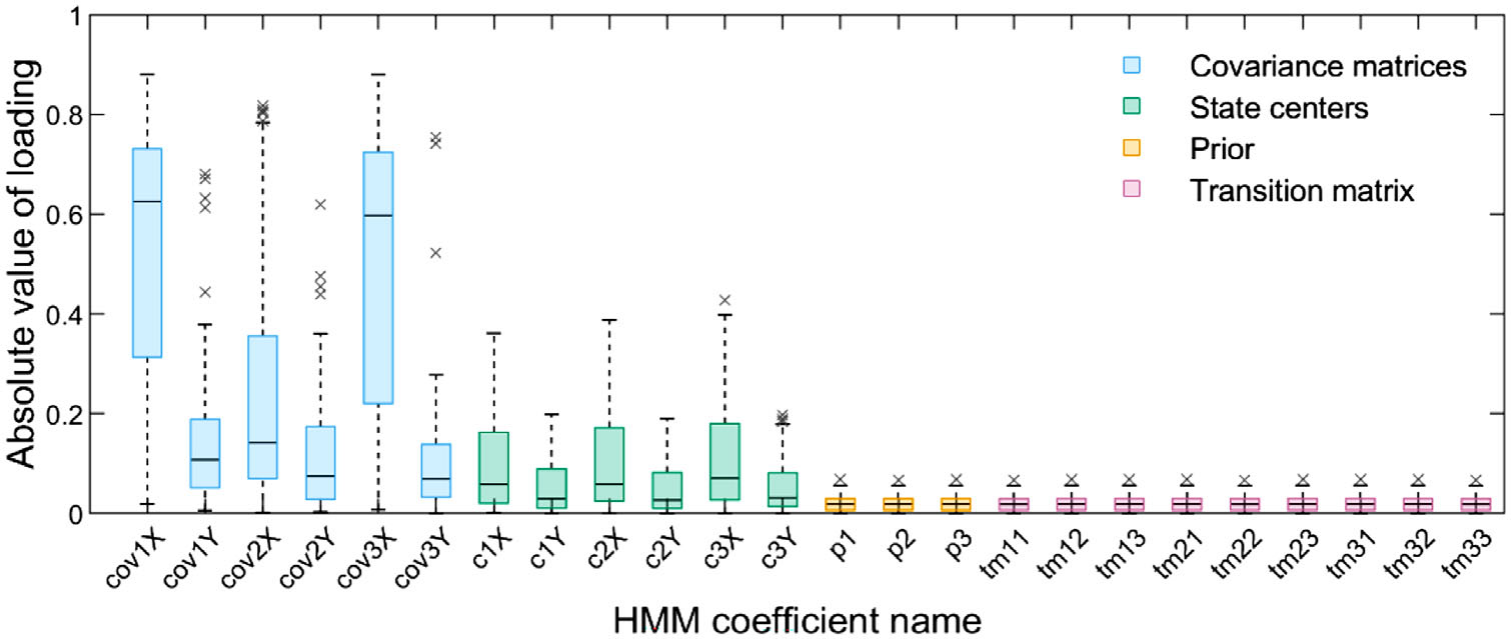
Absolute values of loadings of HMM coefficients in the first principal components. Each boxplot shows the distribution of these values across all critical frames. Colors (see plot legend) encode elements of HMMs to which different coefficients (labelled on the *x*-axis and explained in the text) belong. Note that here, unlike in Figure 2, we treat the centers of ROIs (states) and their covariance matrices as separate elements of HMMs and indicate them with different colors. HMM = hidden Markov model; ROI = region of interest.

Figure 4 shows the absolute values of loadings for each HMM coefficient in the first principal components of all sets of HMMs fitted to the scanpaths (see the Supplemental Material for the analogical plot presenting loadings for all principal components). Examining this Figure provides several insights into the specifics of the gaze patterns we registered. The coefficients of the covariance matrices that determine variance along the horizontal dimension in each ROI (in other words, ROI width) had the highest absolute values of loadings (cov1X, cov2X, and xov3X in the plot). Therefore, the eye movements along this dimension were the aspect of gaze behavior that varied between observers the most, and given that we observed the identical pattern in the loadings from the components from two to seven, it was the primary source of variance in the data. This phenomenon can be considered jointly with the low importance of the state (ROI) centers (c1X, c1Y, c2X, c2Y, c3X, and c3Y). Then, both can be meaningfully interpreted if one considers that frames were wider than taller and that many depicted several characters with heads located at similar heights but spread horizontally. Social information strongly attracts fixations (Birmingham et al., 2008; Cerf et al., 2009; End & Gamer, 2017; Flechsenhar & Gamer, 2017), so most participants likely fixated frame regions showing the heads, which resulted in the low variability in ROI center locations. However, the high variability in ROIs widths indicated the degree to which different participants were shifting their gaze within and around regions occupied by heads or other key scene elements was variable. The direction along which this variability occurred (horizontal) likely resulted from the aspect ratio of the frames and the distribution of content in them.

Next, the low importance of the transition matrix determining the probabilities of transitioning between the ROIs (whose coefficients are marked in the plot as tm11, tm12, and so on) might seem difficult to reconcile with the observation that there was high variability in the eye movements along the horizontal direction, but this is not the case. The states (ROIs) usually covered the locations of multiple fixations, so although the variance resulting from fixating different regions within ROIs was high between observers, the order in which the different ROIs were inspected (and the ROI center locations) could remain relatively stable.

Finally, upon closer inspection of Figure 4, one can notice that cov2X has a lower loading in the first component than cov1X and cov3X. This difference likely stems from the second state usually being in the middle of the frame—it, therefore, captured the initial eye tracker samples that landed on the central frame region overlapping with where the fixation dot preceding each frame was displayed (it captured other samples as well). This issue might also explain the low importance of the prior (probability distribution determining how likely each state is to be fixated first; p1, p2, and p3 in the plot) —it was likely similar between participants because all of them were starting frame exploration from the same central position.

To summarize, the two conditions differed mainly regarding the degree of horizontal spread of the eye movements around the clusters of key elements of the scenes (mainly, but not exclusively, human heads) that were usually aligned along the horizontal direction. This result dovetails with findings from our previous article (Pedziwiatr, Heer et al., 2023), in which we showed that in the discontinuous condition, participants exhibited slightly more exploratory gaze behavior than in the continuous condition. Therefore, while our previous work revealed the nature and direction of the effects of prior knowledge on gaze behavior, here we gained insights into the relationship between these effects and specific image content.

#### Distances-to-boundaries analysis

Finally, we exploited the fact that HMMs holistically describe individual scanpaths—and thereby may capture participant-specific characteristics of eye movements—by investigating if our participants exhibited any idiosyncratic patterns of oculomotor behavior. Specifically, we examined if the strength of the effects of prior knowledge on eye movements was influenced by the individual characteristics of participants. This issue is interesting because the latter scenario would hint at the existence of yet unknown individual differences in the reliance on prior knowledge for gaze guidance. To address issue, we relied on a geometrical interpretation of the classification analysis conducted on the reduced-dimensionality data. According to this interpretation, the classifier creates a plane in a multidimensional space (classification boundary) and points (scanpaths) belonging to different conditions are on each side of the plane. In this distances-to-boundaries analysis, we measured how far from the classification boundary the scanpaths of the individual participants were on average in each condition and then tested if these averages were related between conditions. However, because of our design, these averages were, by necessity, calculated over different sets of critical frames. This fact renders the interpretations of the results—which were statistically significant—challenging. Despite these interpretation difficulties, we believe that our distances-to-boundaries analysis may still hint at underexplored individual differences in gaze control, so we report it in the Supplemental Material.

### Experiment 2: Main results

Recall that our study consisted of two similar experiments, differing with respect to the placement of the attention-check questions. In experiment 1—in which the data presented in all the previous sections was collected—these questions were shown to participants after each critical frame. Given that in the discontinuous condition the critical frames were noticeably different from the context frames preceding them, it is possible that they acted as warning signals about the upcoming questions that prompted participants to adjust their viewing strategy. This adjustment could not be made in the continuous condition, in which the critical frames belonged to the same films as the context frames and were, therefore, inconspicuous. To ensure that the predictability of the questions in one condition but not in the other did not underlie the differences between conditions that we attributed to the relevance of the participants’ prior knowledge to critical frames’ content, we conducted experiment 2. Fifty participants took part in it. This experiment had an identical structure and used identical stimuli as experiment 1 but, crucially, the attention-check questions could appear after any frame (see Pedziwiatr, Heer et al. [2023] for details). Therefore, the occurrence of questions was no longer predictable, and participants did not have the opportunity to adjust their oculomotor behavior in anticipation of them.

We repeated two key analyses reported in previous sections on data from experiment 2. The first was the classification analysis with the dimensionality reduction for different seeds of a random number generator (described in the section HMMs and classification: Caveat two—a random number generator); see Table 2 for the results. In comparison with experiment 1, for experiment 2 fewer outcomes were statistically significant (two vs. seven) and the mean obtained accuracies were lower. Interestingly, when we analyzed metrics of oculomotor behavior calculated for the same data, we found largely similar differences between our conditions as in experiment 1 (Pedziwiatr, Heer et al., 2023). This discrepancy indicates that HMMs are less sensitive to our effects of interest than our previous approach. The second analysis we repeated was the analysis of loadings of the first principal components. Its results were essentially identical as in experiment1 and we include them in the Supplementary Material.

**Table 2.** Results of the same analysis as presented in Table 1 (classification analysis of 10 sets of HMMs differing regarding the seed of a random number generator used when fitting them) for data from experiment 2. IQR = interquartile range; *SD* = standard deviation. Each *p* < 0.05 is printed in bold.

Seed	Mean obtained classification accuracy (*SD*)	Mean chance level classification accuracy (*SD*)	Median obtained classification accuracy (IQR)	Percent of frames classified above chance	*p* value for the mean
0	50.02 (15.46)	49.95 (0.32)	54.55 (8.71)	73.75	0.504
1	48.85 (16.8)	49.92 (0.37)	53.49 (11.82)	70	0.745
2	53.34 (12.76)	49.9 (0.32)	56.32 (7.91)	82.5	**0.017**
3	49.76 (17.37)	49.92 (0.3)	54.55 (7.98)	77.5	0.552
5	49.55 (17.86)	49.94 (0.36)	55.56 (9.31)	72.5	0.605
8	50.7 (14.95)	49.93 (0.34)	54.55 (7.98)	77.5	0.328
13	50.34 (16.58)	49.88 (0.35)	55.05 (10)	77.5	0.398
21	50.05 (17.03)	49.89 (0.32)	55.56 (9.22)	77.5	0.473
34	48.32 (18.02)	49.99 (0.36)	54.55 (9.09)	73.75	0.848
55	52.68 (13.22)	49.83 (0.37)	55.56 (8.04)	81.25	**0.035**

## Discussion

In our study, we investigated how recently accumulated knowledge affects eye movements to natural scenes. We used static film frames arranged in sequences that differed regarding all frames except the last one, called the critical frame. In the continuous condition, frames presented before the critical frame (called the context frames) were the frames preceding it in a film from which it was extracted. In the discontinuous condition, the context frames were extracted from a different film. In consequence, participants always viewed the critical frames after acquiring knowledge of some unfolding events, but, crucially, this knowledge was relevant to the content of the critical frames only in the continuous condition. In our previous article (Pedziwiatr, Heer et al., 2023), we compared several characteristics of gaze behavior between these two conditions and found that in the discontinuous condition, participants’ eye movements were more exploratory than in the continuous condition. Here, we analyzed the same data using a novel approach, complementary to the one we adopted previously, and investigated if the information contained in the spatiotemporal patterns of eye movements was sufficient to distinguish between our conditions. To this end, we fitted a HMM to each scanpath we recorded and, for each critical frame separately, tested how well a simple classifier could assign these HMMs to experimental conditions from which their underlying scanpaths originated. We found that this was the case and, additionally, identified two interesting caveats of the HMMs-based approach. Next, we explored the HMMs fitted to the data and found that horizontal eye movements were likely the feature that was the most important for the classification process (see the Loadings of the first principal components—Analysis and interpretation section for a detailed discussion).

Our study draws from the tradition of typical image viewing studies that involve showing multiple static images to participants in well-controlled laboratory conditions. In the majority of such studies the images are unrelated to each other and therefore, for any given image, the information from images preceding it is irrelevant to its content. In our study, in contrast, the frames were related to each other. Importantly, this relatedness was constituted by the unfolding of depicted events, so it often extended beyond mere similarity in the visual features of successive frames in a sequence. These features often varied from frame to frame substantially because the sequences often included changes in camera position between the frames or changes in the location of events (e.g., a man shown in a car in one frame is shown on the street next to that car in a subsequent frame, indicating that he got out of it).

By using these stimuli, we bridged the gap between studies that largely ignored knowledge accumulation, and at least one branch of research that focused on it: research on visual-narrative understanding. In our previous article we argued that visual-narrative understanding offers a useful framework for interpreting our results (Hutson, Smith, Magliano, & Loschky, 2017; Hutson et al., 2018; Hutson et al., 2022; Loschky et al., 2020; Smith, 2012). It is based on the assumption that accumulating knowledge about events as they unfold leads to the formation of a mental model that is constantly updated with the upcoming information (Loschky, Larson, Smith, & Magliano, 2020). According to this framework, the differences in eye movements we observed between our conditions reflect the fact that in the continuous condition, but not in the discontinuous, information from the critical frame could be incorporated into the current mental model of events. Therefore, these differences demonstrate that the recently accumulated prior knowledge is being taken into account in gaze guidance. Here it must be mentioned that this type of knowledge is not the only type of knowledge that contributes to gaze guidance. Another type of such knowledge is general knowledge about the world and regularities within it, for example, the knowledge about typical contexts in which certain objects appear (e.g., tractors, but not octopuses, are typical for barnyards; Loftus & Mackworth [1978]; see also Coco, Nuthmann, & Dimigen [2020] and, for a review, Võ, Boettcher, & Draschkow, [2019]). How the recently accumulated prior knowledge interacts with it in gaze guidance posits a research questions we intend to pursue in the future.

The effects of prior knowledge—likely underpinning the classification accuracy—were stronger in experiment 1 than in experiment 2. There are two points we would like to make based on these observations. First, it is noteworthy that in our previous analysis of the same data, in which we compared multiple metrics of oculomotor behavior between conditions, we found similar effects in both experiments (Pedziwiatr, Heer et al., 2023). This suggests that the classification analysis, although it facilitates complex data exploration, is less sensitive to the differences between conditions than our previous approach. Second, these quantitative differences between the results of our two experiments likely stem from the differences between the two experimental procedures. In experiment 1, an attention-check question was asked after each critical frame and therefore, in the discontinuous condition only, the critical frames were reliable predictors of the upcoming questions (because only in this condition were these frames markedly different from the preceding frames). This predictability could lead to the anticipation of the questions potentially eliciting an adjustment of eye movements. The presence of this adjustment in one condition, but not in the other, likely led to the higher classification accuracy.

Although the obtained classification accuracy differed between our experiments, it was rather low in both. One factor that might explain this low classifier performance is that critical frames usually contained only several regions strongly attracting fixations (usually human faces). Given that the scope of locations likely to be fixated was limited, the variability in scanpaths—and, in consequence, the variability in the HMMs fitted to them—was limited too. Therefore, the differences between conditions that the classifier aimed to capture had a limited ‘space’ to manifest themselves. An even more important reason why the classification accuracy was rather low is that we did not undertake any steps to maximize it (e.g., we did not test multiple classifiers). This was because accuracy maximization was not our goal. Rather, we wanted to demonstrate the classification analysis as a proof of concept and then use it as a springboard for data exploration.

Nevertheless, although we did not strive to maximize the classification accuracy, we identified two caveats of our approach that influence it. First, we observed that both chance level and obtained classification accuracies depended on the feature-to-sample ratio, that is, the ratio of the number of values describing a single HMM (features) and a number of HMMs being classified (samples). For our raw data, this ratio was high enough to substantially inflate both accuracies. A straightforward way to address this issue was to reduce data dimensionality using principal components analysis. This reduction not only made the interpretation of results easier owing to the alignment of empirically determined and theoretical (intuitively expected) chance levels, but also increased the percent of frames classified above chance. This caveat, known in the machine learning literature (Combrisson & Jerbi, 2015; Vabalas et al., 2019), to our best knowledge has not yet been highlighted in the context of eye-movements modelling.

The second caveat we identified was that the seed of a random number generator used when fitting HMMs to scanpaths can have a profound influence on the results of classification analysis. Repeating this analysis 10 times, each time using HMMs fitted after initializing the random number generator with a different seed, lead to three interesting observations. First, we found that out of several measures of classifier performance we calculated for our set of frames (Table 1), median obtained classification accuracy was the least variable across the 10 repetitions, which suggests that this measure is most robust to changes of the seed. Second, the differences between the highest and the lowest classification accuracies obtained for HMMs fitted using different seeds were often substantial. Therefore, our results suggest that in analytical settings similar to ours, it is advisable to report classification accuracies for a range of random seeds. Our third observation was that the permutation-based statistical test that we used did not yield homogenous results for our 10 different sets of HMMs, which suggests that caution needs to be exercised whenever drawing conclusion from its outcome. These three observations (and the observations we made regarding the first caveat) do not constitute an in-depth analysis of the consequences of the caveats we found. Such analysis, while potentially insightful, is beyond the scope of the present article.

To summarize, we recorded the eye movements of participants viewing static film frames (called the critical frames) in two conditions. These conditions differed regarding the prior knowledge that could be accumulated before viewing these frames. In one condition, this knowledge was relevant to their content, in the other—it was not. We modelled individual scanpaths as HMMs and applied machine learning techniques to analyze them. Although this approach enabled complex data exploration, we found that it was not free from caveats. Nevertheless, and most importantly, it shed light on the underexplored factor contributing to the control of eye movements, namely, recently accumulated prior knowledge.

## Supplementary Material

Supplement 1
