# Supplementary material for: Influence of prior knowledge on eye movements to scenes as revealed by hidden Markov models
Source: J Vis. 2023 Sep 18;23(10):10. doi: 10.1167/jov.23.10.10 (PMC10511023; doi:10.1167/jov.23.10.10)
Supplement: Supplement 1 [file jovi-23-10-10_s001.docx]

Influence of prior knowledge on eye movements to scenes as revealed by hidden Markov models – **Supplemental Material**

Marek A. Pedziwiatr^*^, Sophie Heer, Antoine Coutrot, Peter Bex, Isabelle Mareschal

^*^Corresponding author (marek.pedziwi@gmail.com)

**Distances-to-boundaries analysis**

This exploratory analysis sought to answer whether participants showed distinctive tendencies in their oculomotor behaviors that could provide insights into individual differences in the reliance on prior knowledge for gaze guidance.

**Method.** To investigate this issue, we used the same HMMs as used when interpreting the loadings of the first principal components (see *Loadings of the first principal components* section in the main text) and developed an analysis we call the distances-to-boundaries analysis. It leverages the fact that the classification procedure described in the main text had an intuitive geometrical interpretation. Specifically, each HMM fitted to a scanpath registered on a given critical frame was a single sample (point) in a space having the dimensionality equal to the number of the HMM’s features and the aim of the classifier was to create a plane in this space to separate HMMs originating from the different conditions. Here, for data from each frame separately, we calculated for each participant the Euclidean distance between the HMM fitted to their scanpath (recall that each participant contributed one scanpath per critical frame) and the classification boundary, but only if this scanpath was classified correctly. We then averaged these distances over frames per participant, separately for the two conditions. Thereby, for each participant, we obtained two values (one per condition) of the average distance between the HMMs corresponding to their scanpaths and the classification boundaries. To probe if these distances were related between conditions, we fitted a linear regression model (y = ax + b) to that data using Matlab function *fitlm*. In order to avoid including data points that would have a disproportionately large influence on the results, we restricted this regression analysis to points with leverage values below 0.083. We arrived at that value using a standard approach, that is, by multiplying the number of parameters of the model (two) by two and dividing the results by the number of data points (48).

We repeated this distances-to-boundaries analysis six times, starting with the HMMs with only one principal component retained and each time increasing the number of retained components by one (up to six). We restricted our analyses to the first six components for two reasons. First, they explained almost all the variance in the data (98.52%, see Figure S1, panel F). Second, we wanted to make sure that our results were unaffected by potential issues related to the curse of dimensionality, that is, counterintuitive phenomena that take place in multidimensional spaces (Aggarwal et al., 2001; Domingos, 2012; Verleysen & François, 2005). Finally, note that here, when deciding if a given HMM was classified correctly, we did not conduct a cross-validation but instead relied on a classifier that was trained once, on all the data. This was because we wanted to be able to compare the distances to the boundary between all HMMs within each frame. Cross-validation would render these distances incomparable because it requires retraining the classifier multiple times, and each retraining would change the classification boundary.

**Results.** The results of the six repetitions of the distances-to-boundaries analysis are shown in Figure S1. For five of them, the slopes in the models we fitted had the same directions (were positive), and two of these positive slopes were statistically significant. These slopes indicate that – at least for cases when the classification was correct (recall that here, we consider only correctly classified scanpaths) – for participants for whom the average distance from the boundary was low (or high) in one condition, it was also low (or high) in the other. Importantly, although we found this effect in an exploratory analysis of data from Experiment 1, it was also present – and even stronger – in the data from Experiment 2 (see Figure S2).


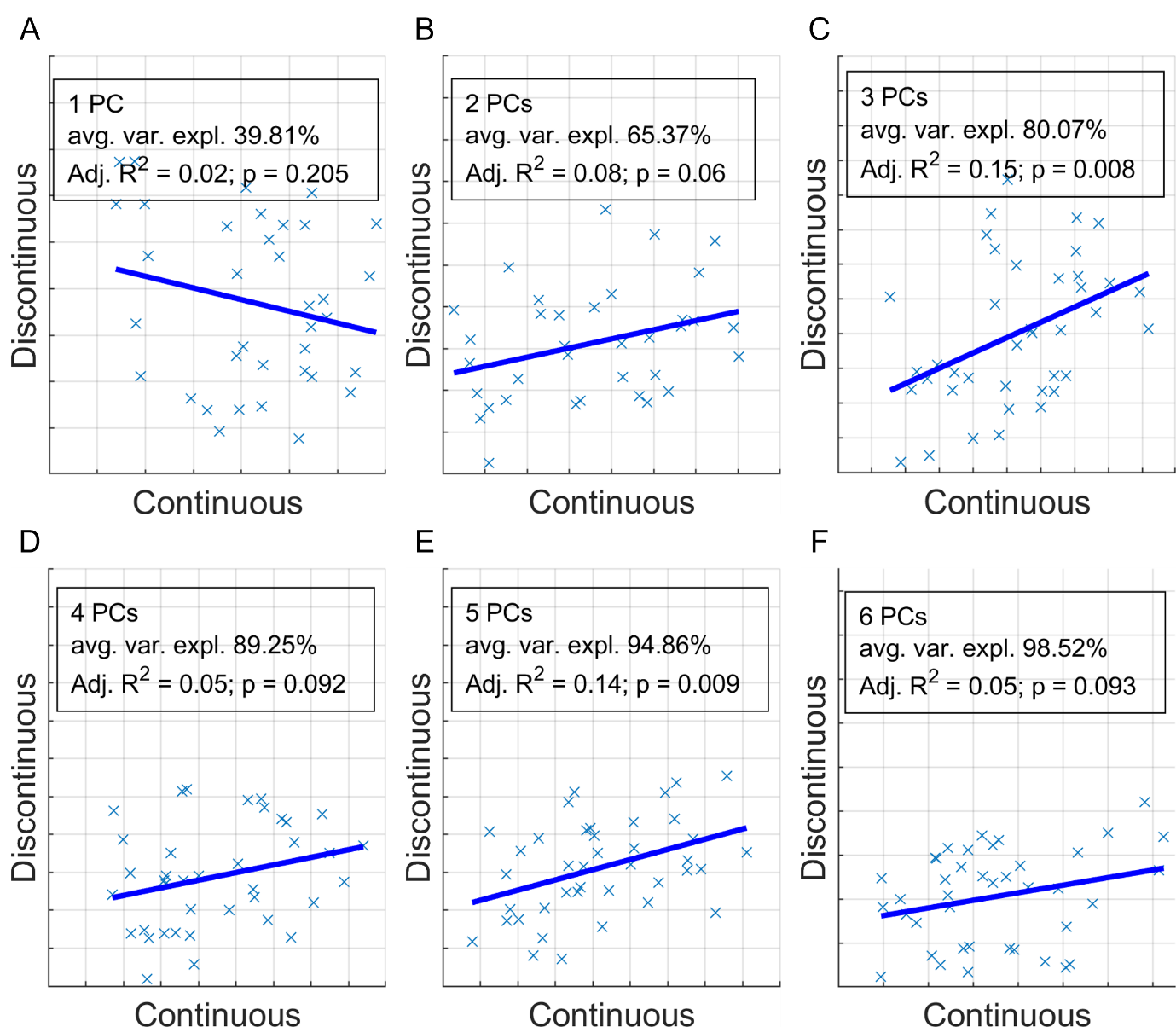


*Figure S1. Results of the distances-to-boundaries analyses. These analyses built on the classification analysis described in the main text and aimed to find indicators of participant-specific patterns of eye movements. All panels show the distances between individual HMMs classified correctly and the boundary established between the two conditions by the classifier. On each panel, blue markers indicate values obtained after averaging these distances over frames per participant per condition, while blue lines indicate linear regression model fit to that data. Subsequent panels show results for data with a different number of principal components retained. Axes on the plots do not have labelled units because these units do not have a meaningful interpretation. However, we ensured that in both axes on each plot, the distance between each two adjacent points is equal and that both axes begin at the same value. Text boxes on the panels provide information about the number of principal components (PCs) retained, the average percent of the variance in the data these components explained, adjusted R^2^ from the linear model, and a p-value for the slope from that model. P-values lower than 0.05 are presented in bold. Finding statistically significant relationships between the per-participant values shown on the panels suggests that our participants exhibited commonalities in oculomotor behavior in different conditions.*


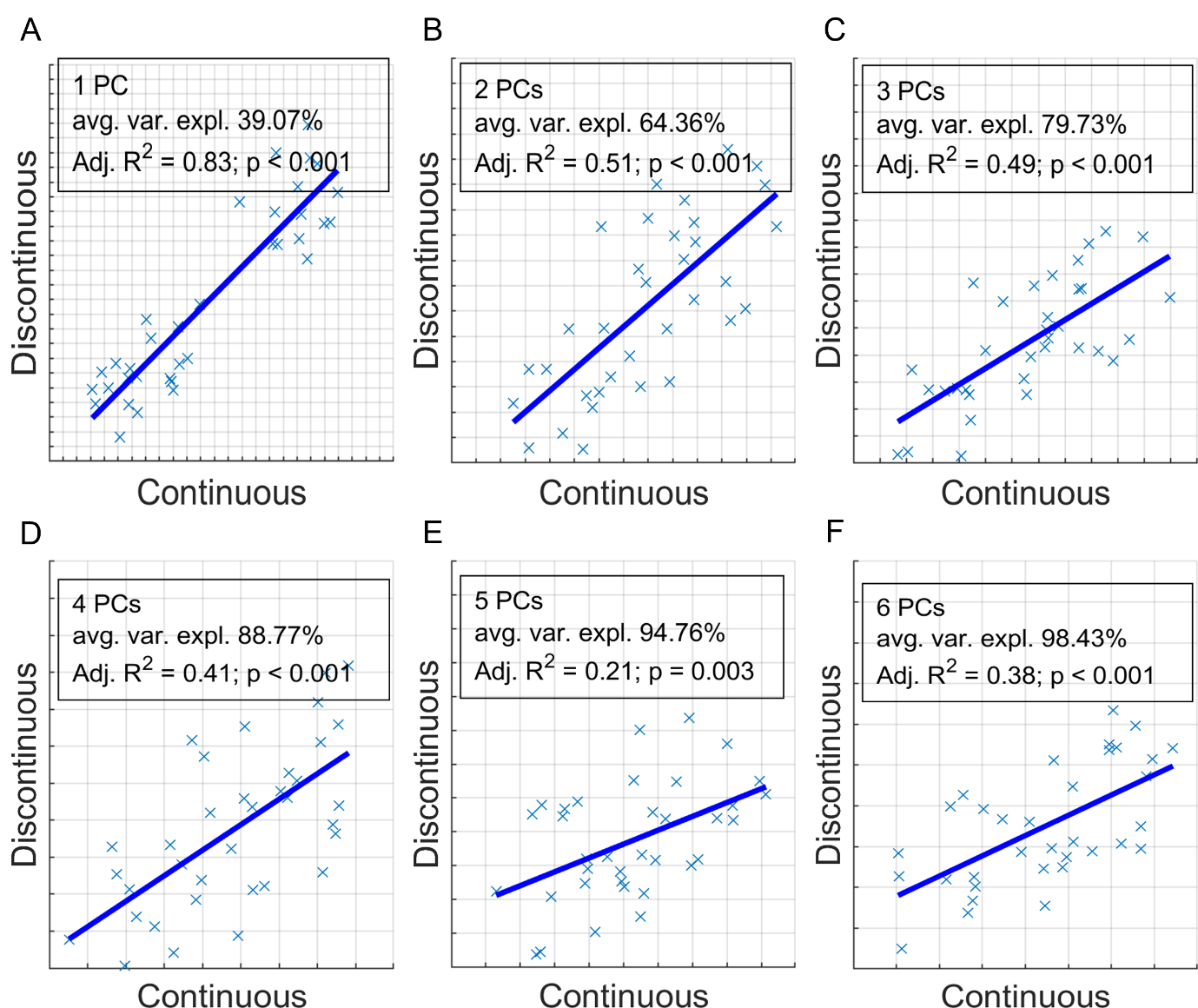


Figure S2. *Results of the same analyses as presented in Figure S1* *(distances-to-boundaries analyses) for data from Experiment 2. See Figure S1 caption for details.*

**Discussion.** While we already knew that the influences of prior knowledge manifested as a difference in gaze behavior in the two conditions, this analysis suggested that the extent to which this difference was evident might be participant-specific. Notably, the indication of the existence of idiosyncratic patterns of gaze behavior we found in both experiments was stronger in Experiment 2 than in Experiment 1. However, in Experiment 2, the obtained classification accuracy was lower. Therefore, while Experiment 1 was more sensitive to the differences between conditions, Experiment 2 was better suited for revealing the individual patterns of gaze behavior. These differences between experiments likely stem from the differences in the predictability of the attention-check questions we discuss in the main text.

Our tentative interpretation of the result reported here assumes that the classifier establishes an ‘objective’ boundary between the conditions. Then, the distance from that boundary measures the ‘extremity’ of oculomotor behavior specific to a given condition. Therefore, some of our participants tended to be systematically more ‘extreme’ in both conditions than others. However, our data provide little insight into the dimension on which this ‘extremity’ is determined. More generally, the fact that each participant viewed different frames in each condition makes comparing oculomotor behavior on a per-participant level challenging. Therefore, we report the results of the distances-to-boundaries analysis not as a new finding per se but as groundwork for future research on individual differences in the effects of prior knowledge on eye movements.

**Loadings of the first principal components – supplemental figures**

Figures S3 and S4 below show the absolute values of loadings of HMMs coefficients in all principal components in data from Experiments 1 and 2, respectively. In each Figure, the numbers on the left-hand side indicate the principal component number. The remaining data presentation details are the same as in Figure 4 in the main text.


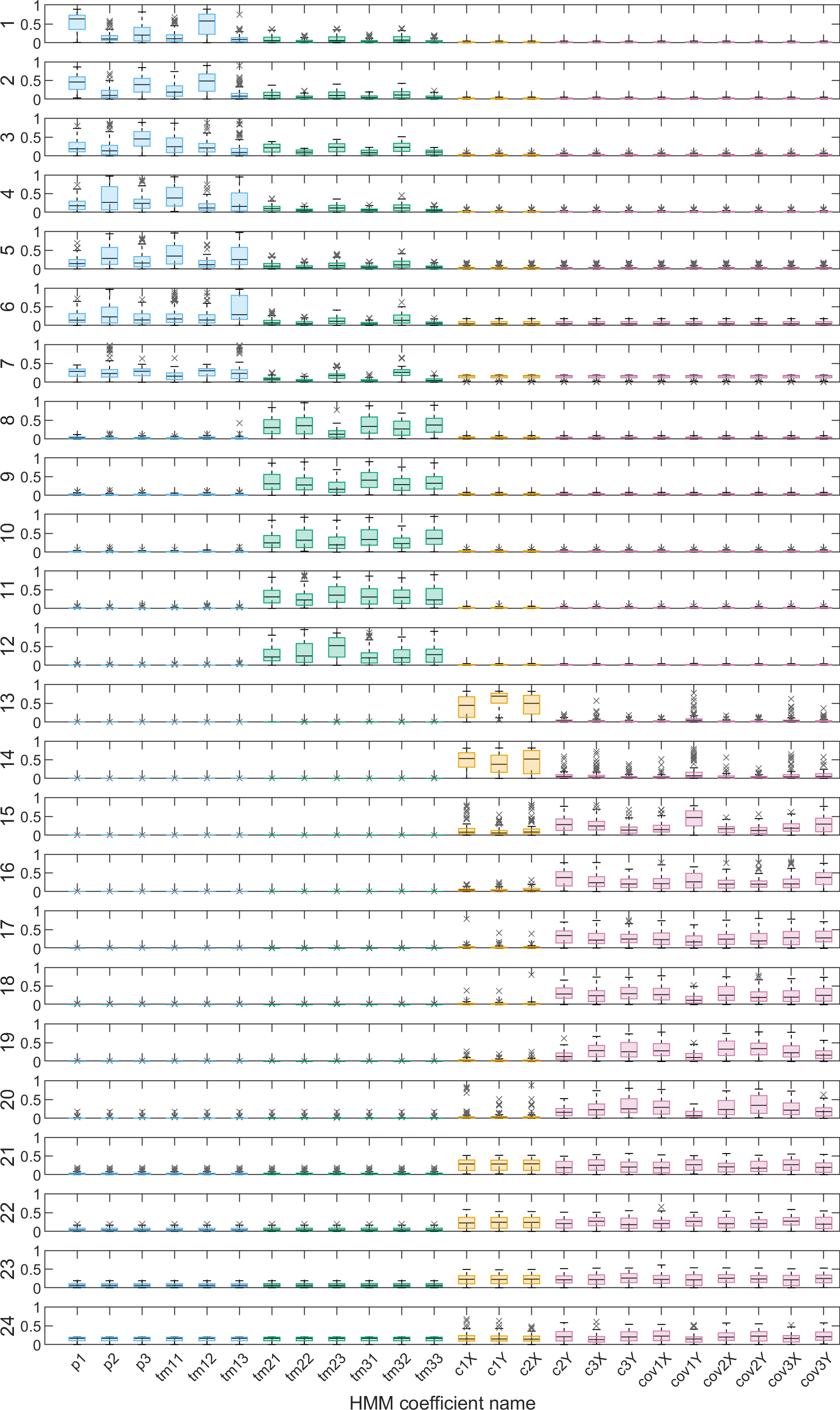


Figure S3. Absolute values of the loadings for data from Experiment 1.


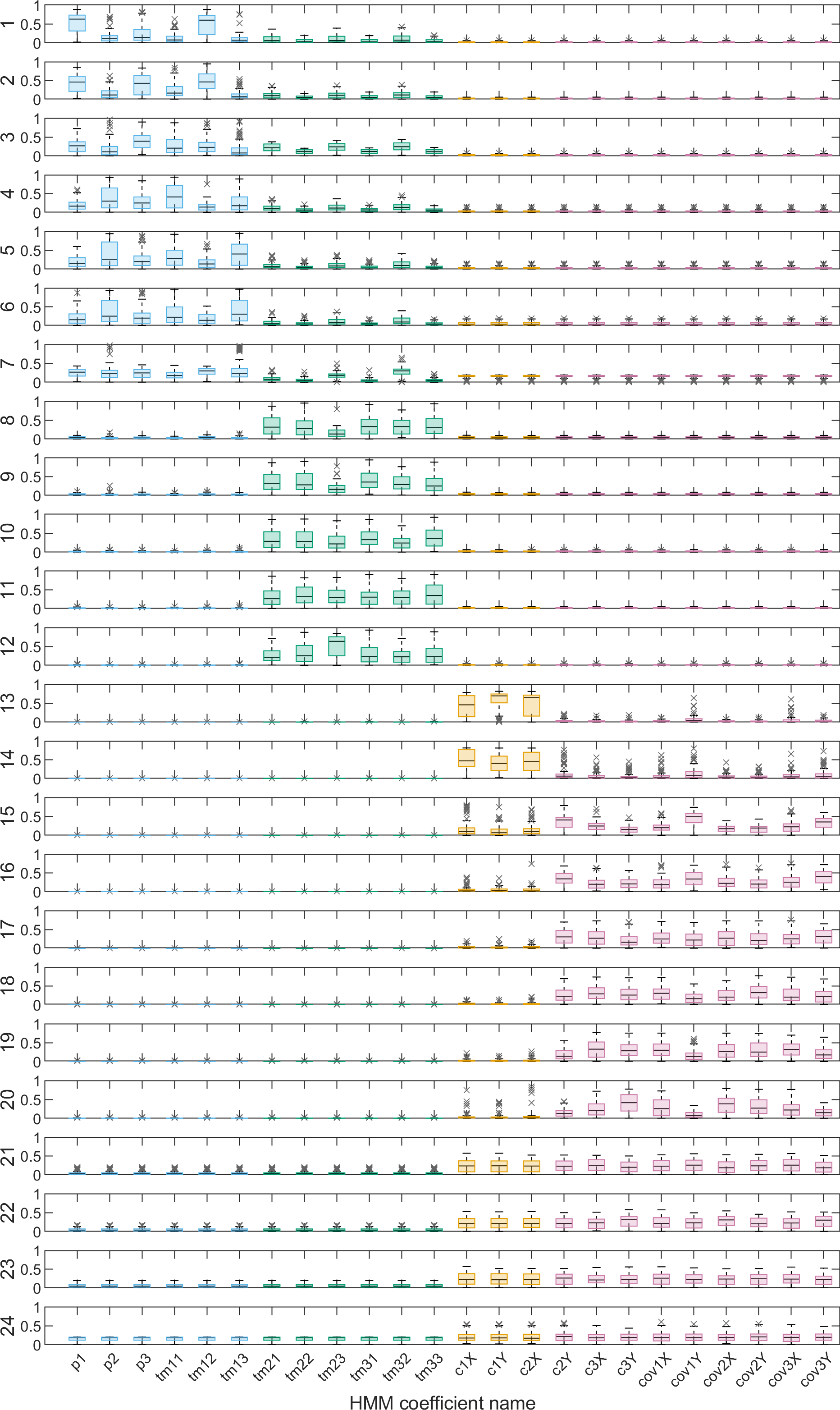


Figure S4. Absolute values of the loadings for data from Experiment 2.

**References**

Aggarwal, C. C., Hinneburg, A., & Keim, D. A. (2001). On the Surprising Behavior of Distance Metrics in High Dimensional Space. In J. Van den Bussche & V. Vianu (Eds.), *Database Theory—ICDT 2001* (Vol. 1973, pp. 420–434). Springer Berlin Heidelberg. https://doi.org/10.1007/3-540-44503-X_27

Domingos, P. (2012). A few useful things to know about machine learning. *Communications of the ACM*, *55*(10), 78–87. https://doi.org/10.1145/2347736.2347755

Verleysen, M., & François, D. (2005). The Curse of Dimensionality in Data Mining and Time Series Prediction. In J. Cabestany, A. Prieto, & F. Sandoval (Eds.), *Computational Intelligence and Bioinspired Systems* (Vol. 3512, pp. 758–770). Springer Berlin Heidelberg. https://doi.org/10.1007/11494669_93
